# Gene expression in the chicken caecum in response to infections with non-typhoid *Salmonella*

**DOI:** 10.1186/s13567-014-0119-2

**Published:** 2014-12-05

**Authors:** Ivan Rychlik, Marta Elsheimer-Matulova, Kamila Kyrova

**Affiliations:** Veterinary Research Institute, Hudcova 70, 621 00 Brno, Czech Republic

## Abstract

Chickens can be infected with *Salmonella enterica* at any time during their life. However, infections within the first hours and days of their life are epidemiologically the most important, as newly hatched chickens are highly sensitive to *Salmonella* infection. *Salmonella* is initially recognized in the chicken caecum by TLR receptors and this recognition is followed by induction of chemokines, cytokines and many effector genes. This results in infiltration of heterophils, macrophages, B- and T-lymphocytes and changes in total gene expression in the caecal *lamina propria*. The highest induction in expression is observed for matrix metalloproteinase 7 (MMP7). Expression of this gene is increased in the chicken caecum over 4000 fold during the first 10 days after the infection of newly hatched chickens. Additional highly inducible genes in the caecum following *S*. Enteritidis infection include immune responsive gene 1 (IRG1), serum amyloid A (SAA), extracellular fatty acid binding protein (ExFABP), serine protease inhibitor (SERPINB10), trappin 6-like (TRAP6), calprotectin (MRP126), mitochondrial ES1 protein homolog (ES1), interferon-induced protein with tetratricopeptide repeats 5 (IFIT5), avidin (AVD) and transglutaminase 4 (TGM4). The induction of expression of these proteins exceeds a factor of 50. Similar induction rates are also observed for chemokines and cytokines such as IL1β, IL6, IL8, IL17, IL18, IL22, IFNγ, AH221 or iNOS. Once the infection is under control, which happens approx. 2 weeks after infection, expression of IgY and IgA increases to facilitate *Salmonella* elimination from the gut lumen. This review outlines the function of individual proteins expressed in chickens after infection with non-typhoid *Salmonella* serovars.

## Table of contents

1. Introduction
2. Early events following *Salmonella* infection of chickens
3. Inflammation
4. Cytokine gene expression in the caecum
5. Cells infiltrating mucosa after *Salmonella* infection
6. Total gene expression in the chicken caecum after *Salmonella* infection
  6.1. Chicken genes down-regulated after *Salmonella* infection
  6.2. Chicken genes induced after *Salmonella* infection
    6.2.1. Inducible genes expressed in cells of non-leukocyte origin
    6.2.2. Inducible genes expressed in both macrophages and heterophils
    6.2.3. Inducible genes expressed in macrophages
    6.2.4. Genes expressed in heterophils
    6.2.5. Inducible genes expressed in B-lymphocytes
    6.2.6. Inducible genes expressed in T-lymphocytes
7. Age dependent response
8. Response of chickens to different serovars
9. Conclusions
10. Competing interests
11. Authors’ contributions
12. Acknowledgements
13. References

## 1. Introduction

Poultry flocks represent the most important reservoir of zoonotic *Salmonella enterica* for humans. *S. enterica* prevalence in poultry varies across different countries but even the most developed countries report around 1% of flocks as *Salmonella* positive. Countries with a more complicated epidemiological situation may report over 10% of flocks as *Salmonella* positive. Even though chickens infected with *S. enterica* usually do not show any gross clinical signs (except for those infected with *S. enterica* serovar Gallinarum and its biovar Pullorum), *Salmonella* is able to persist in the chicken host for a prolonged period of time. Poultry thus becomes a reservoir of this pathogen for humans. However, despite the absence of gross clinical signs, a closer look at the cellular and molecular level reveals extensive interactions between *Salmonella* and the chicken in the caecum. Understanding these interactions can be used for advanced interventions aimed at the reduction of *Salmonella* prevalence in poultry.

## 2. Early events following *Salmonella* infection of chickens

Chickens can be infected with *S. enterica* at any time during their life. However, infections within the first hours and days of their life are epidemiologically the most important, as newly hatched chickens are highly sensitive to *Salmonella* [1-3]. Infection with different *Salmonella* serovars in chickens can be divided into two main groups according to the course of infection. Isolates of serovar Gallinarum and its biovar Pullorum exhibit limited intestinal colonisation and cause little inflammation and, instead, rapidly spread to systemic sites where they continue to replicate. This results in a typhoid course of disease with a high fatality rate [4,5]. The second group consists of all the remaining, non-typhoid serovars of *S. enterica*. A characteristic feature of these serovars is their extensive multiplication in the gut lumen, induction of an inflammatory response in the caecum, but limited spread into deeper tissues such as the liver and spleen associated with only a limited multiplication in these tissues, especially in chickens older than 1 week [1-3]. If the generic term “*Salmonella*” is used in this review, it will refer to non-typhoid serovars, although the majority of information has been obtained for serovars Enteritidis and Typhimurium.

After an initial multiplication in the gut lumen and the adaptation of gene expression to a new environment, *Salmonella* adheres to intestinal epithelial cells. This interaction is dependent on different fimbrial or non-fimbrial adhesins. Up to 13 fimbrial operons with different roles in adhesion to abiotic surfaces or epithelial cells were identified in the genome of *S*. Enteritidis [6] and 12 different fimbrial operons were identified in the genome of *S*. Typhimurium [7]. Additional fimbrial operons found in other *Salmonella* serovars [8] or single nucleotide polymorphisms found within the same fimbrial genes present in different serovars [9] may further affect their adhesion to the chicken gut epithelium.

**Figure 1:**
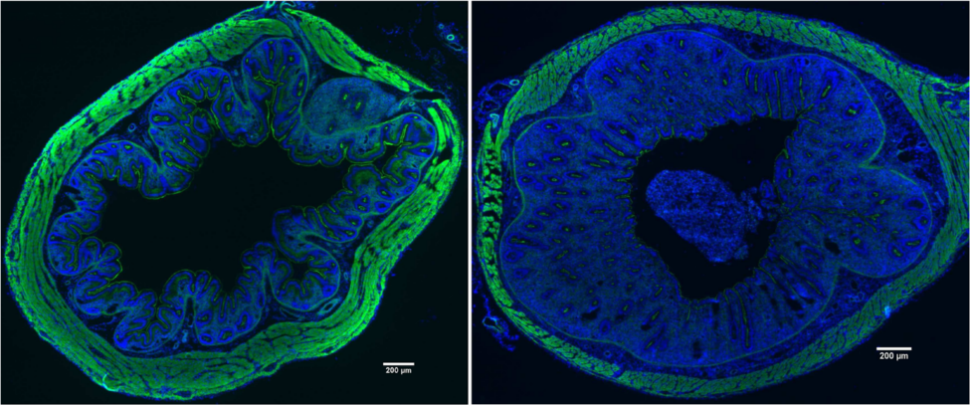
**Caecum morphology with nuclei stained with DAPI (blue colour) and actin stained with phalloidin (green colour).** Left, caecum of a healthy 5-day-old chicken with frequent invaginations. Right, caecum of a 5-day-old chicken infected on day 1 of life with *S*. Enteritidis with mucosal oedema in response to *Salmonella* infection. This figure originates from authors’ unpublished experiments. Bars in each panel indicate 200 μm.

Expression of the type III secretion system encoded by *Salmonella* pathogenicity island 1 (SPI1) is essential for the next step in *Salmonella* colonisation in chickens. Using this secretion system, *Salmonella* irreversibly adheres to the surface of the epithelial cell as shown with HeLa cells [10] and injects its own proteins into the cytosol of epithelial cells. This results in actin cytoskeleton rearrangements, membrane ruffling and finally *Salmonella* uptake [11]. This is the first step during which *Salmonella* is recognised by the chicken host as a pathogen since in the absence of intact SPI1, the inflammatory signalling inducible by *Salmonella* is nearly absent [12]. In other words, the interaction of *Salmonella* with the gut epithelium mediated by fimbrial and non-fimbrial adhesins is not enough to trigger an extensive inflammatory response in vivo. Induction of inflammatory signalling also leads to changes in caecal morphology. The longitudinal and transverse folds with small villi typical for the caecum of healthy chickens are reduced (Figure 1). Instead, inflamed caeca display extensive oedema and a thickened appearance associated with an influx of leukocytes.

Following uptake into the cell, *Salmonella* is present in a membrane-surrounded vacuolar structure. A decrease in both pH and nutrient supply in this environment induces a new expression profile in *Salmonella* which results in the induction of another type III secretion system encoded by *Salmonella* pathogenicity island 2 (SPI2). The major function of this secretion system is to transport *Salmonella* proteins across the vacuolar membrane to the cell cytosol. These proteins interfere with the fusion of the *Salmonella* containing vacuole with lysosomes and its maturation into the phagosome thereby maintaining intracellular survival of *Salmonella* [13]. Although it may appear that the functions of SPI1 and SPI2 encoded type III secretion systems are quite distinct, several papers indicated that the SPI1 encoded type III secretion system is also important for intracellular interactions of *Salmonella* with murine and porcine macrophages [14,15]. Moreover inactivation of either SPI1 or SPI2 type III secretion systems results in similar defects of *S*. Typhimurium or *S*. Enteritidis in the colonisation of the chicken liver and spleen [16,17]. This is in clear contrast with that of mice for which the importance of SPI2 encoded type III secretion system clearly dominates over the requirement of the SPI1 encoded type III secretion system [18].

The early interactions of *Salmonella* with the host are mainly *Salmonella* driven. Initial attachment by fimbrial and non-fimbrial adhesins is followed by permanent docking of *Salmonella* on the surfaces of epithelial cells. This is achieved by penetration of the type III secretion system apparatus through the cytoplasmic membrane of both *Salmonella* and the host cell. Injection of *Salmonella* secreted proteins results in *Salmonella* uptake and intracellular appearance in the vacuole like structure.

## 3. Inflammation

Invasion of the gut epithelium followed by *Salmonella* interaction with chicken macrophages and heterophils triggers a chicken immune response. Initial *Salmonella* recognition is based on sensing a subset of SPI-1 translocated proteins which activate Rho-family GTPases to promote bacterial invasion. Excessive stimulation of Rho-family GTPases activates the transcription factor nuclear factor kappa-light-chain-enhancer of activated B cells (NF-κB) which results in proinflammatory cytokine expression [19]. Interestingly, the inflammatory signalling can be separated from the invasion in human epithelial cells [20]. SPI1 encoded proteins can influence inflammation also directly, both stimulating [21] as well as suppressing [22] the inflammatory response in human cell lines. The chicken’s response to *Salmonella* infection is further developed by toll-like receptor (TLR) sensing. Although TLR2, TLR4, TLR5, TLR15 and TLR21 each contribute to *Salmonella* recognition [23,24], the key TLR in *Salmonella* sensing are TLR4 and TLR5, whose ligands are lipopolysaccharide (LPS) and flagellin, respectively. Following TLR4 and TLR5 ligand binding, heterophils induce IL-1β, IL-6, and IL-8 expression [25,26]. Monocytes sense LPS via TLR4 signalling to induce iNOS and NO radical production, cytokines and the effector genes described below. As NO radicals are produced by iNOS from arginine with ornithine as a reaction by-product, enzymes allowing recycling of ornithine back to arginine, i.e. argininosuccinate synthase (ASS1) and argininosuccinate lyase (ASL1), are also induced in inflamed tissues and in chicken monocytes and macrophages following *Salmonella* infection [12].

The absence of flagellin expression in serovar Gallinarum and biovar Pullorum has also been used to explain their lower recognition by the chicken immune system and their easier spread to systemic sites [27]. Experiments with genetically modified *S*. Gallinarum or *S*. Typhimurium confirmed the importance of flagellin sensing by TLR5 during the *Salmonella*-chicken host interaction [4,28]. On the contrary, *Salmonella* flagellin also acts as an antigen stimulating antibody production. Such antibodies may bind to flagellin during secondary infection and interfere with TLR5 sensing [29]. This phenomenon can be of extra benefit if aflagellated attenuated vaccines are used for chicken immunisation. These may induce a specific immune response to all *Salmonella* antigens except for flagellin. A challenge wild type, i.e. flagellated, strain is then recognised by both the adaptive immune system and the TLR5 dependent innate immune system [30].

Initial recognition of *Salmonella* by epithelial cells and resident leukocytes results in chemokine and cytokine signalling. Although the cytokine signalling can be induced by the type III secretion system proteins alone, the inflammatory process is exacerbated following the intracellular presence of *Salmonella* and recognition of LPS and flagellin by TLR4 and TLR5.

## 4. Cytokine gene expression in the caecum

Following the initial sensing of *Salmonella* by epithelial cells and resident lymphocytes, macrophages and heterophils, an orchestrated process aimed at restricting the spread of *Salmonella* to deeper tissues is triggered. The induction of cytokines and immune relevant proteins such as IL1β, IL6, IL8, IL12, IL17, IL18, IL22, IL23, IFNγ, LITAF or iNOS following *Salmonella* infection of chickens have been reported repeatedly in many studies [1,31,32]. The major function of signalling molecules is to attract additional leukocytes from the circulation to the site of infection (IL1β, IL8, IL17) [33,34], to increase general resistance of epithelial cells to the infection (IL22) [35], or to stimulate macrophages for NO radical production and *Salmonella* inactivation (IFNγ) [36].

IFNγ, IL17 and IL22 are cytokines inducible in different T-lymphocytes and their expression may vary according to the vaccination status of infected chickens [37,38]. IL1β, IL6, IL8, and IL18 are characteristic of chicken macrophages [37]. LITAF and iNOS are expressed in all chicken leukocyte subpopulations with a minor inducible effect in macrophages and all other lymphocyte subpopulations after intravenous infection with *S*. Enteritidis [38].

In addition to protein cytokines and chemokines, non-protein prostaglandin signalling is associated with *Salmonella* infection in the chicken caecum as well. Chicken heterophils exposed to *S*. Typhimurium flagellin in vitro produced prostaglandin E2 [39]. Moreover, prostaglandin D2 synthase (PGDS) is induced and prostaglandin D dehydrogenase (HPGD), an enzyme involved in prostaglandin D2 degradation, is suppressed in the caecum of chickens orally infected with *S*. Enteritidis [12]. This may lead to prostaglandin D2 accumulation and the progression of an inflammatory response.

## 5. Cells infiltrating mucosa after *Salmonella* infection

In the absence of infection, chicken heterophils represent numerically the most abundant leukocyte population in the caecal *lamina propria* followed by macrophages and T-lymphocytes. B-lymphocytes are nearly absent from the caecal *lamina propria* of chickens up to the age of 10 days [33] and, consequently, immunoglobulin transcripts are not detected in the chicken caecum during the first week of life [12]. After *Salmonella* infection, the increase in the population of heterophils in the caecum is the lowest when compared with other leukocyte subpopulations. Heterophils gradually increased from day 2, reached their maximum on day 4, slightly decreased on day 6 but remained elevated till day 10 [33]. The infiltration of macrophages is the fastest and most time restricted reaching its maximal short-peak infiltration 2 days post-infection and returning back to basal levels approx. 6 days after the infection of newly hatched chickens. In comparison to macrophages, T-lymphocyte infiltration remains elevated for approx. 2-4 days longer, i.e. until days 8-10 of life, similar to heterophils [33]. B-lymphocytes exhibit the highest change in their counts in the caecal *lamina propria* but their numerical increase is considerably affected by the absence of B-lymphocytes in the caecal *lamina propria* during the first week of life and the fact that *Salmonella* infection stimulates the formation of B-lymphocyte follicles [33]. Interestingly, when leukocyte infiltrates were determined in the jejunum of chickens, neither macrophages nor CD4 and γδ T-lymphocytes increased following oral infection of newly hatched chickens with *Salmonella* and the only population which infiltrated the jejunum in response to *Salmonella* infection was represented by CD8 T-lymphocytes [40].

Induction of cytokines or increased synthesis of prostaglandins leads to changes in gene expression in resident cells and leukocytes trafficking to the site of infection from circulation. Infiltration of caeca with macrophages, heterophils, and T- and B-lymphocyte causes changes in the total gene expression. This results in a control of infection 2-3 weeks after inoculation of newly hatched chickens with *Salmonella*.

## 6. Total gene expression in the chicken caecum after *Salmonella* infection

**Figure 2:**
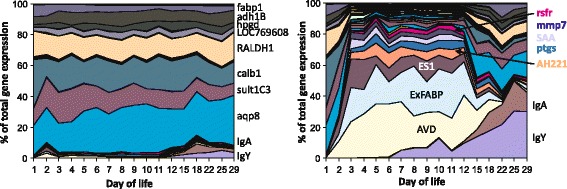
**Gene expression in the chicken caecum following oral infection with** ***S*** **. Enteritidis on the day of hatching.** Chickens were infected on the day of hatching and expression of 45 selected genes in the chicken caecum was determined by real-time PCR including the expression in the age-matched, non-infected controls. Left panel, gene expression in the non-infected chickens, mind the increase in the expression of IgY and IgA in the second week of life. Right panel, gene expression in the caecum of infected chickens, mind the dramatic changes in the total caecal expression within 48 h after infection and also an increase in IgY and IgA expression during the recovery phase. For more details see reference [13].

Changes in gene expression in the entire tissue can be detected by real-time PCR, western blot or ELISA. However, all these techniques require the selection of target genes or proteins to be characterised which introduces a bias into such studies. The bias can be overcome by the use of genome-wide techniques such as RNA/cDNA microarray, RNA/cDNA next generation sequencing and protein mass spectrometry. These techniques have enabled the identification of many new genes and proteins not yet associated with the chicken’s response to *Salmonella* infection [12,40] (Figure 2, Table 1). Such proteins, both positively and negatively correlating with infection, will be introduced in the following paragraphs.

**Table 1:** **List of genes induced or suppressed in the chicken caecum 4 days after infection of newly hatched chickens with** ***Salmonella*** **Enteritidis**

**Gene**	**Name**	**Function**	**Cell**	**Protein**	**RNA**	**Ref.**
GENES UPREGULATED IN THE CHICKEN CECUM						
MMP7	matrix metallopeptidase 7	degradation of extracellular matrix proteins	Ep	ND	1430.8	[12]
MUC2L	mucin-2-like	mucus production	Ep	ND	4.02	[12]
IFIT5	interferon-induced protein with tetratricopeptide repeats 5	uncapped RNA binding and inactivation	unknown	ND	6.75	[12]
ES1	ES1 protein homolog	unknown	unknown	25.8	28.30	[12]
IL8	interleukin 8	chemokine	Ep, M	ND	5.36	[12]
iNOS	inducible NO synthase	NO radical production using arginine as a substrate	M, T, B	ND	37.09	[12]
ExFABP	extracellular fatty acid binding protein	fatty acid and bacterial siderophore binding	Ht, M	ND	151.6	[12]
MRP126	MRP-126, S100A9, calprotectin, calgranulin	calcium and zinc binding	Ht, M	312	42.57	[12]
SERPINB10	serpin peptidase inhibitor	protection of tissues against own proteases	Ht, M	1313	30.95	[12]
TRAP6	trappin 6-like	protection of tissues against own proteases	Ht, M	ND	36.46	[37]
IRG1	immune responsive gene 1	itaconic acid and reactive oxygen species production	Ht, M	ND	83.17	[37]
SAA	serum amyloid A	acute phase protein, LPS binding	Ht, M	ND	84.63	[37]
C1QA	complement C1a component	complement	Ht, M	ND	3.23	[37]
C1QB	complement C1b component	complement	Ht, M	ND	1.28	[37]
C1QC	complement C1c component	complement	Ht, M	ND	3.00	[37]
C3	complement 3	complement	Ht, M	81.4	10.78	[12]
AVD	avidin	biotin binding, tissue reparation	M	ND	15.15	[37]
ASL2	argininosuccinate lyase	arginine recycling	M	12.2	4.36	[12]
IL1B	interleukin 1 β	cytokine	M	ND	28.09	[12,32,38]
IL18	interleukin 18	cytokine	M	ND	5.39	[32,38]
CATHL2	cathelicidin-2	antimicrobial peptide	Ht	317	2.15	[12]
CATHL3	cathelicidin-3	antimicrobial peptide	Ht	ND	1.50	[12]
GAL1	gallinacin-1	antimicrobial peptide	Ht	ND	0.70	[32]
GAL2	gallinacin-2	antimicrobial peptide	Ht	ND	1.22	[32]
LYG2L	lysozyme G-like 2	antimicrobial peptide	Ht	283	37.21	[12]
PGDS	prostaglandin D2 synthase	prostaglandin D2 synthesis	Ht	ND	10.42	[12]
RSFR	leukocyte ribonuclease A-2	angiogenesis, antimicrobial peptide, ribonuclease	Ht	915	5.70	[12]
TGM3	transglutaminase 3	protein cross-linking	Ht	643	2.76	[12]
IgY	immunoglobulin Y heavy chain	antigen binding	B	ND	8.51	[37]
IgA	immunoglobulin A heavy chain	antigen binding	B	ND	3.50	[37]
IgM	immunoglobulin M heavy chain	antigen binding	B	ND	3.91	[37]
IgL	immunoglobulin light chain	antigen binding	B	ND	7.09	[37]
TGM4	glutamine γ-glutamyltransferase 4	protein crosslinking	B, M	484	24.63	[12]
IL17	interleukin 17	cytokine	T	ND	5.43	[12,32,38]
IL22	interleukin 22	cytokine	T	ND	63.18	[12,32,38]
IFNG	interferon γ	cytokine	T	ND	32.08	[12,32,38]
NKL	NK-lysin	lysis of own aberant cells	T	ND	16.73	unpubl.
GENES DOWNREGULATED IN THE CHICKEN CAECUM						
HPGD	hydroxyprostaglandin dehydrogenase 15-(NAD)	inactivation of prostaglandin D2	unknown	ND	4.0	[12]
LOC769608	uncharacterized oxidoreductase	unknown	unknown	2.77	4.3	[12]
ALDOB	aldolase B, fructose-bisphosphate	unknown	unknown	2.64	4.6	[12]
CALB1	calbindin 1	calcium transport	Ep	2.26	5.5	[12]
SULT1C3	sulfotransferase	solubilasation and detoxification	unknown	ND	6.2	[12]
RALDH1	aldehyde dehydrogenase 1	unknown	unknown	2.74	7.9	[12]
FABP1	fatty acid binding protein 1	fatty acid transport	Ep	2.92	8.3	[12]
AQP8	aquaporin 8	water, ammonia and hydrogen peroxide transport	Ep	ND	9.9	[12]
ADH1B	alcohol dehydrogenase 1B	unknown	unknown	4.52	13.2	[12]

Ep, epithelial cell, Ht – heterophil, M – macrophage, B – B lymphocyte, T – T lymphocyte, protein – fold induction or suppression determined by protein mass spectrometry, RNA – fold induction or suppression determined by real-time PCR, ND – not determined.

### 6.1. Chicken genes down-regulated after *Salmonella* infection

Infection of chickens with *Salmonella* is usually characterised by the induction of an inflammatory response and an increased expression of particular genes. However, there are also genes, whose expression decreases after *Salmonella* infection. Following oral infection of 5 month old hens with *S.* Enteritidis, 32 different genes were downregulated in the liver 10 days post-infection [41]. These genes belonged mainly to two functional categories; either common metabolic functions or cell cycle control. The fold suppression in the liver was quite low, mostly around a factor of 2 [41]. In addition, 9 different genes were identified, whose expression transiently decreased in the caecum in response to *S*. Enteritidis infection of newly hatched chickens [12]. None of the genes suppressed in the liver were identical with those suppressed in the caecum. However, similar to the observations in the liver, most of the genes suppressed in the caecum were involved in normal gut function such as nutrient or electrolyte transport, or detoxification of certain substrates. Suppression of the genes in the caecum was higher than that in the liver and sometimes approached a factor of 100. The genes with the most reproducible suppression included aquaporin 8 (AQP8), calbindin 1 (CALB1), fatty acid binding protein 1 (FABP1), sulfotransferase 1C (SULT1C3) or 15-hydroxyprostaglandin dehydrogenase (HPGD). Although genes encoding basal gut functions decreased in expression, these markers of *Salmonella* infection are not as sensitive as the genes coding for chemokines and cytokines. We recently described that the caecum has a “buffering” capacity, i.e. the expression of genes involved in normal gut function is preserved even during minor inflammatory response. As long as the expression of inflammatory genes does not increase more than tenfold, the expression of genes like AQP8, CALB1, FABP1, SULT1C3 or HPGD does not decrease. Normal gut functions become temporarily suppressed only when the expression of inflammatory genes continues to increase above a factor of 10 [42].

### 6.2. Chicken genes induced after *Salmonella* infection

Recent progress in genome-wide technologies enabled the identification of many new genes/proteins, which are upregulated in the chicken caecum following *Salmonella* infection. Though many of these proteins have been reported as inducible during different inflammatory disorders of both infectious and non-infectious origin, the function of these proteins in chicken response is less clear. These proteins include effector ones expressed to directly inactivate pathogens or proteins protecting the chicken’s own tissues against damaging itself. A detailed understanding of the function of these proteins in the chicken’s defence against *Salmonella* infection may allow for new applications. One can speculate that it might be possible to administer these proteins, their inhibitors or ligands therapeutically to increase the chicken’s resistance to *Salmonella* infection or other pathogens. These proteins can also be used as part of a vaccine formula to improve their efficacy, similar to the effect of adjuvants. Finally, even without a detailed understanding of their function, the expression of these proteins can be used as markers of inflammation caused by *Salmonella* infection or vaccination [43]. The latter possibility might be more accurate and preferred to cytokine gene expression as these genes are expressed at high levels in the caecum and can therefore be detected and quantified easier than cytokines expressed at low levels.

The most inducible genes in the caecum following *S*. Enteritidis infection include matrix metalloproteinase 7 (MMP7), immune responsive gene 1 (IRG1), serum amyloid A (SAA), extracellular fatty acid binding protein (ExFABP), serine protease inhibitor (SERPINB10), trappin 6-like (TRAP6), calprotectin (MRP126), mitochondrial ES1 protein homolog (ES1), interferon-induced protein with tetratricopeptide repeats 5 (IFIT5), avidin (AVD) and transglutaminase 4 (TGM4). The induction of these proteins commonly exceeds a factor of 50 [12], similar to inductions of chemokines and cytokines such as IL-1β, IL-6, IL-8, IL-17, IL-18, IL-22, IFNγ, AH221 or iNOS [12,31].

#### 6.2.1. Inducible genes expressed in cells of non-leukocyte origin

Genes and proteins highly inducible in the chicken caecum after *S*. Enteritidis infection but not expressed in leukocytes include MMP7, ES1-like protein and IFIT5 [12].

The highest induction in the chicken caecum following *Salmonella* infection was observed for MMP7. Expression of this gene increases over 4000 fold during the first 10 days after the infection of newly hatched chickens and by far exceeds the inductions of all the remaining inducible genes. The function of MMP7 is to degrade extracellular matrix proteins [44]. In humans, MMP7 is expressed by colonic epithelial adenoma cells [45] or colonic epithelial cell line after contact with *E. coli* [46]. Interestingly, this induction could be suppressed by mannose which interferes with type I, mannose-sensitive fimbria produced by *E. coli*. It is worth mentioning that *S*. Gallinarum expresses a structurally different FimH protein which results in *S*. Gallinarum type I fimbria being mannose resistant [9]. This may contribute to less efficient adhesion to epithelial cells, less frequent invasion, lower intracellular presence and consequently, less extensive inflammation in the chicken caecum following infection with *S*. Gallinarum. Although cells expressing MMP7 in chickens have not been identified, MMP7 was not induced in the spleen following intravenous infection and leukocyte subpopulations were also not responsible for its expression in the caecum (unpublished observations). Its expression in response to *Salmonella* infection is therefore specific to the caecum [12] although we do not exclude other mucosal surfaces.

The mitochondrial-like ES1 protein homolog is induced both in the jejunum and caecum of chickens after *Salmonella* infection [12,47]. The ES1 protein homolog is encoded by LOC422305 on chicken chromosome 4. Later annotations designated this gene as *elbB* for enhancing lycopene biosynthesis protein 2, or *yhbL* (isoprenoid biosynthesis protein with amidotransferase-like domain). Protein domain searches also indicate the presence of a type 1 glutamine amidotransferase (GATase1)-like domain but whether this is of any relevance for its induction in the chicken caecum after *Salmonella* infection is completely unknown.

IFIT5 is a type I interferon inducible gene in the human promyelocytic leukemia cell line [48]. Human IFIT5 binds cap free 5′ppp mRNA characteristic of genomic RNA of negative stranded RNA viruses such as the influenza virus [49]. Consistent with this, when the chicken cell line was transfected with the duck RIG gene, which is a pathogen recognition receptor absent in chickens, IFIT5 increased in response to infection with avian influenza virus [50]. IFIT5 can also bind to the host’s own tRNA and in this way interfere with the efficiency of translation eventually leading to the induction of apoptosis [51]. This mechanism might be effective in defence against *Salmonella. Salmonella* infected cells may increase IFIT5 levels and undergo apoptosis or pyroptosis which would result in the release of intracellular *Salmonella* making it available for phagocytosis by cells of the immune system.

*Salmonella* clearance might also be enhanced by the expression of mucin2-like protein which is inducible in the chicken jejunum and caecum following *Salmonella* infection [12,52]. Muc2 protein is expressed by goblet cells and its function is to interfere with *Salmonella* adherence to epithelial cells allowing peristaltis to remove *Salmonella* from the gut lumen.

The cells that are not part of the immune system contribute significantly to the control of *Salmonella* infection. A simple but effective measure is the increase in mucin expression preventing *Salmonella* association with epithelial cells and its clearance by peristaltis. MMP7 activity results in tissue relaxation enabling penetration of leukocytes to the site of infection. Induction of cell death by IFIT5 may result in a release of *Salmonella* from invaded non-professional phagocytes making it available to macrophages and heterophils.

#### 6.2.2. Inducible genes expressed in both macrophages and heterophils

Genes and proteins expressed both in macrophages and heterophils, i.e. specific to phagocytic cells, include IRG1, ExFABP, TRAP6-like gene, SERPINB10, MRP126, SAA and serum complement proteins.

IRG1 was characterised as an LPS-inducible gene in murine RAW macrophages [53]. It is also expressed in chicken macrophages [37]. This protein is localised in the mitochondria of human macrophages or expressed in the murine uterus where it catalyses the synthesis of itaconic acid through the decarboxylation of cis-aconitate, a tricarboxylic acid cycle intermediate. IRG1 also stimulates macrophages to form reactive oxygen species as a by-product of mitochondrial β-oxidation of fatty acids in mice and zebra fish [54,55]. IRG1 may therefore enhance antimicrobial activities of the host by the production of oxidative species.

ExFABP, also known as p20k, lipocallin Q83, ch21 or LCN8, is expressed in chicken macrophages and heterophils [37]. ExFABP is encoded on chicken chromosome 17, just adjacent to lipocalin-15-like and prostaglandin D2 synthase genes (PGDS), the latter gene is also inducible following *S*. Enteritidis infection. Chicken ExFABP was first characterized as a protein capable of binding unsaturated fatty acids with an unknown role in chondrocyte development [56]. In parallel, ExFABP was characterised as an LPS-inducible, acute phase protein [56,57]. ExFABP also stimulates cell proliferation and its suppression results in apoptosis [57]. All of this shows that ExFABP plays an important role in tissue repair and/or differentiation in the absence of any infection. However, chicken ExFABP and quail lipocalin Q83 have dual binding capacities because besides the fatty acid binding, they can also bind bacterial siderophores [58]. This makes ExFABP functionally similar to its murine ortholog Lcn2 [59]. In agreement, chicken ExFABP inhibits the growth of *E. coli* in iron-limited media in vitro [58]. Based on the enterochelin binding capacity of Lcn2 in mice [59], it is also possible that ExFABP may not bind glycosylated enterochelin produced by *Salmonella* and *Salmonella* can therefore have a growth advantage over the rest of the caecal microbiota. However, fatty acid binding activity may also be important for chicken defence against *Salmonella* infection. As chickens do not code for myeloperoxidase, production of oxidative species by chicken heterophils is lower than in mammalian neutrophils [60,61]. Alternative pathways may therefore act in chickens and one of these might be dependent on IRG1. Since IRG1-dependent production of reactive oxygen species is positively affected by levels of acetyl-CoA derived from the β-oxidation of fatty acids [54,55], ExFABP might be important for fatty acid transport followed by generating reactive oxygen species during respiration. Interestingly, neutrophil phagosomes in humans were found to contain elevated levels of mitochondrial proteins [62]. Although there might be several explanations for this observation, one of them is that phagosomes may fuse with mitochondria producing reactive oxygen species as a by-product of respiration. Such oxygen species released into the developing phagosome may contribute to pathogen inactivation.

The TRAP6-like protein locus (LOC428141) is located on chromosome 20 and in the current assembly release it can be found as LOC101752219 and *Gallus gallus* sodium/potassium ATPase inhibitor (SPAI-2-like). Trappin-6 has never been studied in chickens experimentally and its identification was based only on sequence similarities [63]. Its likely function is the protection of the host’s own tissues from degradation by its own proteases released by neutrophils such as neutrophil elastase or proteinase 3 [64]. Release of protease inhibitors also reduces inflammatory signalling by neutrophils and thus indirectly protects the host’s own tissue against extensively damaging itself [65]. Trappin-6 is expressed by macrophages [37] and our unpublished data show that it was also highly transcribed in heterophils, but not in lymphocytes. This may further support the hypothesis that its function is to protect the host tissue against its own proteases released during pathogen degradation. Trappins including trappin6-like protein contain transglutaminase substrate domain (GQDPVK consensus sequence) in the N terminal part of the protein [66]. This domain is used by transglutaminases for covalent attachment of trappins to the host’s own tissues as has been shown for elafin, a human ortholog of trappin-6 [67]. Whether transglutaminases TGM3 and TGM4, which are also induced during inflammation [12,37], are responsible for cross-linking of trappin-6 to extracellular matrix proteins has never been studied, however, their simultaneous expression with trappins is suggestive of this. In addition to trappin, SERPINB10 (SERine PRotease INhibitor) is another protein which is induced in the chicken caecum after *Salmonella* infection and which protects host tissues against its own proteases. SERPINB10 is encoded on chicken chromosome 2, forming a cluster with other protease inhibitor genes. SERPINB10 belongs to clade 10 of serpin protease inhibitors and is structurally similar to ovalbumin.

MRP126 (also known as calprotectin, calgranulin or S100A9) is common to chicken, murine and human macrophages and granulocytes [37,68,69]. Calprotectin binds Ca^2+^ and Zn^2+^ [69-71]. Even though its role in the chicken’s response to *Salmonella* infection is largely unknown, it has shown antibacterial effects against both Gram positive and Gram negative bacteria including *Salmonella* [69,72]. On the contrary, Liu et al. showed that *S*. Typhimurium tolerates the presence of calprotectin in mice and thus has a growth advantage over other microbiota present in the gut [71]. The expression of calprotectin was also increased in Peyer’s patches of pigs orally infected with *S*. Choleraesuis [73], or mice infected with *S*. Typhimurium [71].

Serum amyloid A (SAA) is another acute phase protein inducible in response to different infections including *Salmonella* infections. However, its role in defence against *Salmonella* infection is unclear. SAA decreases oxidative burst in leukocytes, likely due to LPS binding [74]. However, serum amyloid A is also induced after viral infections without any LPS stimulus [75]. Heterophils followed by macrophages seem to be its main producers in chickens. Neither T-, nor B-lymphocytes expressed this protein [37]. Serum amyloid A may deposit in joints of chickens resulting in amyloidosis and arthropathy. Some papers associated these disorders with the inflammation induced by vaccines containing strong adjuvants [76]. Consequently, one may speculate that inflammation caused by vaccinations as well as that induced by *Salmonella* infection may predispose chickens to disorders currently understood as production associated diseases.

There are other defence proteins, whose expression increase 2-10 fold and which play an important role in the defence against *Salmonella*. Serum complement proteins are induced in response to *Salmonella* infection. C1q, C1r and C1s proteins binding to LPS-antibody complex are induced in the spleen after intravenous *Salmonella* infection and C3 protein is induced in the caecum following oral infection [12,37].

Macrophages and heterophils are key cells involved in the innate response to *Salmonella* infection. Proteins expressed in both cell types include those involved in LPS neutralisation (SAA and serum complement proteins) and protecting the host tissue against damaging itself (TRAP6-like, SERPINB10). IRG1 increases production of reactive oxygen species and ExFABP and MRP126 restrict bacterial growth by decreasing availability of extracellular Fe^2+^, Ca^2+^ and Zn^2+^.

#### 6.2.3. Inducible genes expressed in macrophages

Avidin is one of a few genes which are exclusively expressed in chicken macrophages. Besides biotin binding, avidin was shown to block chondrocyte proliferation without any effect on their differentiation [77]. Avidin is inducible by LPS and avidin itself can induce ExFABP expression [77]. It is common in egg white and is traditionally associated with its antimicrobial activity [78]. However at least in LB broth it did not exhibit antimicrobial activities against *S*. Enteritidis at concentrations as high as 2.5 mg/mL [37]. This, of course, does not exclude that other bacterial species are sensitive to biotin deprivation by avidin allowing *Salmonella* to get a growth advantage in inflamed intestine over other microbiota members, as has been similarly proposed for Lcn2 binding bacterial siderophore in mice [59]. However, at least two research groups showed that microbiota changes in chickens infected with *Salmonella* are not as dramatic as one would expect [79,80]. A more likely function of avidin therefore might be the restoration of the host’s damaged tissues as avidin affects cell differentiation.

#### 6.2.4. Genes expressed in heterophils

Induction of multiple proteins can be easily recorded following *Salmonella* infection of chickens though their induction is difficult to confirm at the RNA level. Even some genes which have been already mentioned exhibit a great variability in induction in the caecum determined at the protein and mRNA levels. MRP126 was induced in the chicken caecum 312× when determined at the protein level by mass spectrometry but only 42× when determined at the mRNA level by real-time PCR. Additional examples include SERPINB10 (1313 fold induction by protein mass spectrometry but only 31 fold induction determined by real-time PCR) or lysozyme *g*2 LYG2 (283/37) [12]. The most likely explanation is that the induction at the level of transcription is caused by the induction of these genes in macrophages. However, since these proteins are expressed and stored also in heterophil granules, tissue infiltration of heterophils contributes only to the increase at protein level. Altogether this provides an explanation for the discrepancy in induction at the protein and mRNA levels at the site of inflammation.

Heterophils are responsible for pathogen inactivation by the release of two classes of antimicrobial peptides, i.e. cathelicidins CATHL1, CATHL2 (318× protein induction/2× mRNA induction), CATHL3 and gallinacins GAL1, GAL2 and GAL7 (also called avian β-defensins AvBD1, AvBD2 and AvBD7). Since these proteins are present in the granules of chicken heterophils not tightly associated with gene transcription, contradicting reports can be found on their modified expression in response to *Salmonella* infection if real-time PCR is used [32,37,81]. Other proteins which were identified as highly inducible in the chicken caecum at the protein level but almost not inducible at the mRNA level include ribonuclease A homolog (RSFR) and transglutaminase TGM3.

RSFR (915× protein induction in the caecum/6× mRNA induction, see [12]) is characteristic of granulocytes in both chickens and humans [82-84]. This protein exhibits multiple enzymatic activities. It is a ribonuclease A with angiogenic and bactericidal properties. Its ribonuclease function can be separated from its bactericidal activities [83]. RSFR has been shown to have angiogenic potential allowing the restoration of damaged tissues following inflammation [82]. In addition, not only the RFSR protein but also peptides generated by partial digestion of mature RFSR have been shown to have bactericidal effects and a modulatory effect on dendritic cells polarising the immune response towards a Th2 response in chickens and humans [82-84]. RSFR therefore contributes to both tissue reparation and clearance of residual bacterial pathogens.

TGM3 transglutaminase is expressed only in chicken heterophils (644× protein induction/3× mRNA induction). TGM3 was more than 1000 fold induced in the lungs of pigs experimentally infected with *S*. Choleraesuis [85]. Due to its expression in heterophils, it has been discussed above as potentially involved in cross-linking trappin6-like protease inhibitor to host cells. However, it can also be involved in cross-linking other proteins such as fibrin during wound healing, although this would have to be determined in chickens experimentally.

Although chicken heterophils do not express myeloperoxidase and are therefore disabled in pathogen inactivation by reactive oxygen species, they are still able to express lysozyme and antimicrobial peptides of two classes of antimicrobial peptides, i.e. cathelicidins and gallinacins. Besides pathogen inactivation, chicken heterophils are also involved in tissue protection and wound healing by the expression of RSFR, TGM3 and protease inhibitors TRAP6 and SERPINB10.

#### 6.2.5. Inducible genes expressed in B-lymphocytes

In chickens, transglutaminase TGM4 is expressed in B-lymphocytes and to a lesser extent in macrophages [37]. This is rather surprising as a mammalian TGM4 ortholog was found to be expressed exclusively in the prostate. Transglutaminases are commonly expressed by both epithelial cells and lymphocytes in inflamed rat intestinal tract [86]. Interestingly, transglutaminase inhibitor cystamine reduced the inflammation induced by 2,4,6-trinitrobenzene sulfonic acid in rats [86]. Transglutaminases catalyse the formation of an isopetide bond between the carboxyamide group of glutamine and the ε amino group of lysine leading to protein cross-linking. The cross-linking may happen within two amino acid residues of the same protein making it resistant to proteolytic degradation or between amino acids of different proteins [67]. The transglutaminase-dependent cross-linking has also been described in the complex of IgA and soluble CD89 and its interaction with the TfRI transferrin receptor [87]. Although TGM2 was reported as involved in this type of cross-linking, TGM4 in chickens may have a similar function to this one, e.g*.* cross-linking of immunoglobulin opsonised antigens to CD89 or Fc receptors present on the surface of macrophages, heterophils or dendritic cells. Though speculative, this would explain the simultaneous expression of immunoglobulins and TGM4 in B-lymphocytes.

The second group of proteins expressed in B-lymphocytes are immunoglobulins, although transcripts of these genes can also be detected in T-lymphocytes [37]. Following *Salmonella* infection, transcription of IgY and IgM dominate over IgA in the spleen while IgY and IgA dominate over IgM in the caecum [12,37]. Expression of IgY in the caecum can be detected from day 7 of life and IgA was first detected from day 10 of life [12,88], consistent with a gradual influx of B lymphocytes to the caecal *lamina propria* [33]. Additionally, chickens younger than one week did not respond to immunisation with BSA by antibody production [89]. *Salmonella* infection stimulates B-lymphocyte homing to caecal mucosa so that the IgY and IgA transcripts can be detected for the first time approx. 2 days earlier than in non-infected chickens [12,88]. Chickens therefore respond to *Salmonella* infection by antibody production, although this response is not considered as paramount to protection [90]. Mucosal expression of immunoglobulins might therefore be important for pathogen clearance from the intestinal tract during the recovery phase of infection [91], and not for preventing systemic spread of *Salmonella* nor for developing specific systemic immune response.

B-lymphocytes and antibody production are usually of lower importance for chicken resistance to *Salmonella* infection. However, B-lymphocytes infiltrate the site of infection and induce expression of antibodies and TGM4. IgA antibodies may interfere with *Salmonella* attachment to epithelial cells thereby allowing peristaltis to remove *Salmonella* from the gut lumen. Expression of IgY and TGM4 may increase *Salmonella* recognition in deeper tissues, cross-linking antibodies with other soluble serum proteins or cell receptors and more efficient phagocytosis and development of specific immune response.

#### 6.2.6. Inducible genes expressed in T-lymphocytes

The contribution of T-lymphocytes to the total expression in the caecum during primary exposure to *Salmonella* is quite low, although their counts increase after *S*. Typhimurium infection [92]. This does not mean that these cells do not respond to *Salmonella* infection as IL17, IL22 or IFNγ expressed by T-lymphocytes are induced within the range of 10 to 100 fold [38,93]. The only gene which is expressed at high levels that can be easily detected in the caeca is the gene encoding NK-lysin. Hong et al. reported that chicken CD8 and CD4 T-lymphocytes can express NK-lysin [94], similar to our proteomic analyses which show that CD8 and γδ T-lymphocytes are the main sources of NK-lysin in chickens (unpublished observations). As γδ T-lymphocytes can be CD8 positive [95], findings on CD8 and γδ T-lymphocyte expression of NK-lysin may point to the same leukocyte subpopulation.

T-lymphocytes are important for the development of adaptive immune response. Their role during the acute phase of primary infection is limited to NK-lysin expression and combating *Salmonella* infected cells. Intracellular *Salmonella* is then released and becomes available for inactivation by macrophages and heterophils.

## 7. Age dependent response

The chicken response to *Salmonella* infection described so far is easily reproducible in young chickens up to 1 week of age [1-3]. However, the resistance of chickens to *Salmonella* infection increases with age and the response of 6-week-old chickens to *Salmonella* challenge sometimes does not significantly differ from the expression in non-infected control chickens. Neither gene expression, nor leukocyte populations in the lymphoid tissues, change following infection of older birds with *S*. Enteritidis [43,96,97]. This causes difficulties in vaccine testing considering the first vaccination in newly hatched chickens, revaccination 3 weeks later and challenge an additional 3 weeks later, i.e. in 6 week old chickens. Age dependent resistance of chickens to *Salmonella* infection is far from being understood, although it is generally accepted that i) the gut immune system is not fully maturated at the time of hatching [98], ii) the gut *lamina propria* of newly hatched chickens is poorly populated by leukocytes and has yet to be infiltrated by macrophages, heterophils, B- and T-lymphocytes within the first 10 days of life [33], and iii) gut microbiota with a protective effect is not fully developed [99]. Experimental outcomes from germ-free chickens would be valuable in addressing the development of the gut immune system in the absence of microbiota. Similarly, experiments with chickens associated with just one bacterial species would enable us to understand the role of individual bacterial species present in normal chicken gut microbiota and their interactions with the gut immune system, nutrient digestion and the corresponding host expression in caecal tissue.

## 8. Response of chickens to different serovars

There are over 2500 different *Salmonella* serovars, most of them belonging to *Salmonella enterica* subspecies *enterica*. There are major differences in the responses of chickens to serovar Gallinarum and its biovar Pullorum, compared to the rest of the serovars. Infection of chickens with *S*. Gallinarum and Pullorum usually results in lower colonisation of the caecum and spread into the liver and spleen. Similarly, the *S*. Typhimurium replicated faster than *S*. Choleraesuis in the intestinal wall of pigs and faster replication of *S*. Typhimurium was associated with higher induction of the proinflammatory cytokines [100]. The low counts in the caecum together with differential flagella and fimbria expression described above explain the low levels of inflammation induced by the typhoid serovars.

All other serovars are capable of inducing an inflammatory response in the chicken caecum following oral infection in newly hatched chickens [31,42]. Invasion is dependent on the type III secretion system localised on pathogenicity island 1 which is a trigger for the chicken immune response since SPI1 mutants are unable to induce inflammation [12]. This also indicates that strains which are either defective in or at least have a decreased ability to invade chicken epithelial cells may cause a lower or no inflammatory response. It can therefore be hypothesised that less invasive serovars will induce a lower inflammatory response [31]. However, there are differences in invasiveness among isolates belonging to the same serovar. Consequently, we have recently infected chickens with several different isolates of *S*. Enteritidis, Typhimurium and Infantis showing that strain selection may considerably affect the outcome which might be completely discordant with the expected properties of the serovar [42].

## 9. Conclusions

**Figure 3:**
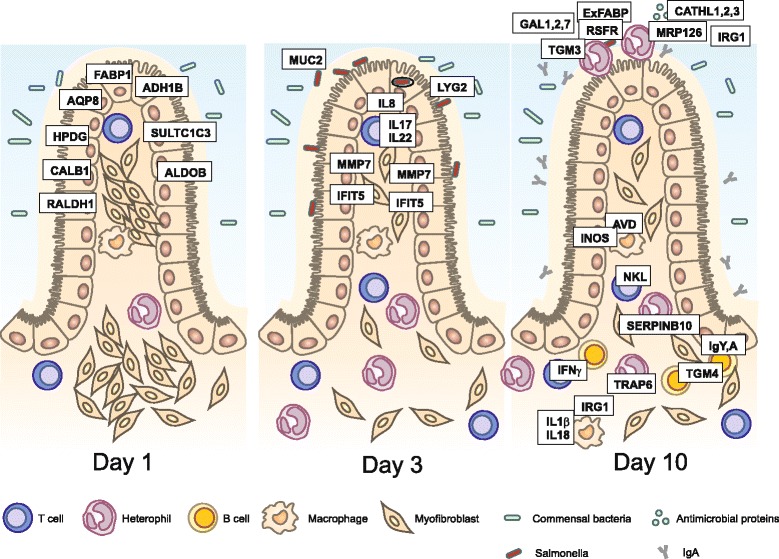
**Gene expression in the chicken caecum following oral infection of newly hatched chickens.** Genes dominantly expressed in the caecum of non-infected chickens are those associated with normal gut function, i.e. nutrient uptake. The first genes induced within 48 hours after the infection represent components of the innate immune system and chemokines. Induction of MMP7 results in tissue relaxation allowing for the infiltration of leukocytes. B cells are absent in the chicken caecum until day 7 of life, irrespective of infection. Ten days post-infection, cytokines modifying immune responses are expressed by T cells and macrophages, and heterophils, macrophages and B cells express proteins that facilitate *Salmonella* clearance. Heterophils furthermore express proteins (TRAP6 and SERPINB10) that protect chicken tissue against its own proteases. Proteins shown in the figure are placed close to the cell type which is the most important for their expression.

Events in the chicken caecum following infection of young chickens with non-typhoid serovars can be summarised as follows. After oral ingestion of *Salmonella*, its multiplication in the gut lumen and invasion into the epithelial cells, IL8 and IL17 cytokine signalling appears initially. In parallel, IFIT5, LYG2 and MMP7 are induced, with IFIT5 and MMP7 being expressed by cells of non-leukocyte origin, i.e*.* this response is independent of infiltrating leukocytes. Maximal expression of these genes is achieved within 48 hours post-infection of newly hatched chickens with *S*. Enteritidis. Inflammatory signalling is then maintained by the expression of IL1β, IL18, IL22 and IFNγ. These cytokines reach their maximal expression approx. 4 days post infection. Expression of this set of cytokines is accompanied by a high expression of effector proteins such as IRG1, ExFABP, iNOS, AVD, TRAP6, SERPINB10, TGM4 and TGM3 (Figure 3). Once the infection is under control, which happens approx. 2 weeks after infection, expression of IgY and IgA increases to facilitate *Salmonella* elimination from the gut lumen [12].

As summarised in the previous paragraph, *Salmonella* can induce an inflammatory response in the caecum of newly hatched chickens, but the resistance of chickens older than one month is quite high. What happens during the first month of life in the chicken caecum in the absence of any infection? What happens in the caecum of germ free chickens? What is the influence of microbiota colonisation on the development of the chicken immune system? Does chicken microbiota act directly on *Salmonella*? What are the most effective components of chicken microbiota against *Salmonella*? All of these questions are interesting and without any clear answer. Moreover, all of these questions can nowadays be relatively easily addressed using new instruments in the area of mass spectrometry and/or NextGen sequencing. All of this guarantees that the interaction between *Salmonella* and chickens will remain an attractive model for future studies. Such studies will allow for new interventions in *Salmonella*-chicken infection as well as measures which could be applied to other avian species and other infections.
